# Genome-wide scan on plasma triglyceride and high density lipoprotein cholesterol levels, accounting for the effects of correlated quantitative phenotypes

**DOI:** 10.1186/1471-2156-4-S1-S47

**Published:** 2003-12-31

**Authors:** Jing-Ping Lin

**Affiliations:** 1 Office of Biostatistics Research, DECA, National Heart, Lung and Blood Institute, National Institutes of Health, 6701 Rockledge Drive, Bethesda, Maryland, USA

## Abstract

**Background:**

Plasma triglyceride and high density lipoprotein cholesterol levels are inversely correlated and both are genetically related. Two correlated traits may be influenced both by shared and unshared genes. The power to detect unshared trait-specific genes may be increased by incorporating correlated traits as covariates. The power to localize the shared genes may be improved by bivariate analysis. Univariate genome scans were carried out on triglyceride (high density lipoprotein cholesterol) with and without using high density lipoprotein cholesterol (triglyceride) as a covariate, and bivariate linkage analysis on triglyceride and high density lipoprotein cholesterol using the 330 Framingham pedigrees of the Genetic Analysis Workshop 13 data. The results of five genome scans were compared to determine the chromosomal regions which may harbor the genes influencing variation specific to triglycerides, specific to high density lipoprotein cholesterol, or the covariation of both triglyceride and high density lipoprotein cholesterol.

**Results:**

The results of our five genome scans identified some chromosomal regions with possible quantitative trait loci (QTL) that may specifically influence one trait, such as the regions on chromosome 1 (at 1 cM near marker 280we5), on high density lipoprotein cholesterol, or control the covariation of both traits, such as the regions on chromosome 7 (at 169 cM near marker GATA30D09), chromosome 12 (at 3 cM near marker GATA4H03), chromosome 20 (at 49 cM near marker GATA29F06), chromosome 2 (at 146 cM near marker GATA8H05), and chromosome 6 (at 148 cM near marker GATA184A08) on triglyceride and high density lipoprotein cholesterol. The one on chromosome 6 had a LOD score of 3.1 with the bivariate linkage analysis.

**Conclusion:**

There is strong evidence for a QTL on chromosome 6 near marker GATA184A08 appearing to influence the variation of high density lipoprotein cholesterol and triglycerides in the Framingham population.

## Background

Genetic components play important roles in determining serum lipid levels including plasma triglyceride (TG) and high density lipoprotein cholesterol (HDL-C) levels. Studies demonstrate that both TG and HDL-C concentrations are significantly related to cardiovascular diseases [1,2]. There is an inverse correlation between TG levels and HDL-C levels [3,4]. The genetic correlation between TG and HDL-C is higher compared with environmental correlation. Therefore, the metabolism or genetic backgrounds of HDL-C and TG may be interrelated. Two correlated traits could be under the influence of shared genes, pleiotropy, as well as being influenced by unshared genes [5]. Bivariate linkage analysis is capable of improving both the power and localization of the shared gene for correlated quantitative traits [6]. A study by Arya et al. showed that the power to detect the unshared genes for a trait using univariate linkage analysis could be greatly increased by incorporating other correlated traits as covariates [7]. On the other hand, the power to detect chromosomal regions harboring shared genes in univariate linkage analysis may decrease when the corrected trait is incorporated as a covariate. Here, univariate genome-wide linkage analyses were performed on TG with and without HDL-C as a covariate, on HDL-C with and without TG as a covariate, as well as bivariate linkage analysis on TG and HDL-C. The results of these five genome scans were compared to identify the chromosomal regions where trait specific genes for TG, or for HDL-C, or shared genes to both traits might reside.

## Methods

### Study population

The real Framingham data (Genetic Analysis Workshop 13 (GAW13), problem 1) were used in this study. The Framingham Heart Study began in 1948 with the recruitment of 5209 residents, 2336 men and 2873 women aged 28–62 years, from Framingham, Massachusetts. The subjects have undergone biennial examinations since the study began. In 1971, the Framingham Offspring Study was initiated, in part, to evaluate the genetic components of cardiovascular disease etiology. In total, there were 5124 subjects aged 5–70 years recruited. The offspring subjects have been examined every 4 years (except in the first two examinations with 8 years intervening). Our study population included the 330 largest, extended Framingham families with a total of 1702 genotyped individuals.

TG, HDL-C, and the other phenotypes used in these analyses were measured at the original cohort examinations 10–12 (TG and HDL-C were measured once for each individual over the three examinations) and first offspring cohort examination. Both cohorts were measured almost at the same time, in the early 1970s. The measurement of these phenotypes has been detailed elsewhere [4].

### Statistical analysis

Variance-component univariate linkage analysis implemented in SOLAR (1.7.4) [8] was used for heritability estimation, and two-point and multipoint linkage analyses. This method is based on the assumption of a multivariate normal distribution for the traits tested, and a violation of the assumption may result in inflated type I error rates [9,10]. Since TG and HDL-C are not normally distributed, with skewness and kurtosis being 2.8 and 14.0 for TG and 0.3 and 1.5 for HDL-C, respectively, logTG and logHDL-C were used in the analysis. The covariates selected and incorporated by SOLAR in the heritability estimation and linkage analysis included age, sex, smoking, and alcohol consumption. For two separate genome scans logHDL-C (logTG) was incorporated as a covariate on logTG (logHDL-C). For the bivariate linkage analyses, a new version of SOLAR (2.0.1) used the same covariates as in the univariate linkage analysis. In this version, bivariate LOD scores are reported with 1 degree of freedom. These equivalent LOD scores are comparable to univariate LOD scores.

## Results and Discussion

The total number of individuals measured for TG, HDL-C as well as all covariates used for the heritability estimates and linkage analyses (age, sex, smoking, and alcohol consumption) was 1999. The correlation between logTG and logHDL-C was -0.4 (*p *< 0.01).

The heritability estimates were 44 ± 5% and 40 ± 5% for logTG and logHDL-C, respectively, after covariate adjustment. The chromosomal regions of the five genome scans with a maximum multipoint LOD score equal or greater than 1.5 are summarized in Table 1.

Scans 1 and 2 were genome scans on logTG with and without logHDL-C as a covariate. On scan 1, four regions on chromosomes 6, 7, 12, and 20 were identified with maximum multipoint LOD scores equal or great than 1.5. When logHDL-C was added as an additional covariate in scan 2, the peak multipoint LOD score decreased for all four chromosome regions. This result may imply that those four chromosome regions harbor genes that control both traits. However, except for the region on chromosome 6, these genes might play more important roles in controlling TG variation than that of HDL-C, since no strong signal was detected on the HDL-C genome scans (scans 3 and 4), and the LOD scores for bivariate analyses were all decreased as well. No new chromosomal region was identified with a LOD score reaching 1.5 by scan 2.

Our scan 1 was similar to a genome scan of logTG in the Framingham Heart Study [4]. The two regions on chromosome 7 and 20 identified by scan 1 were also identified by their study. In addition, a new region on chromosome 6 was identified with a maximum LOD score of 1.7. Basically, we used the same data. However, because only a limited number of variables from the Framingham data were provided to GAW13, our study did not include as many covariates in the analyses as in theirs. In contrast, we did not include body mass index (BMI) as a covariate, because we considered BMI as a correlated trait of both TG and HDL-C, and to include it as a covariate may decrease the power to detect a shared gene effect. Interestingly, the results of our other genome scans provided evidence that the chromosome 6 region may harbor a gene controlling both TG and HDL-C, the same region that was identified by a previous genome scan for BMI [11].

**Figure 1:**
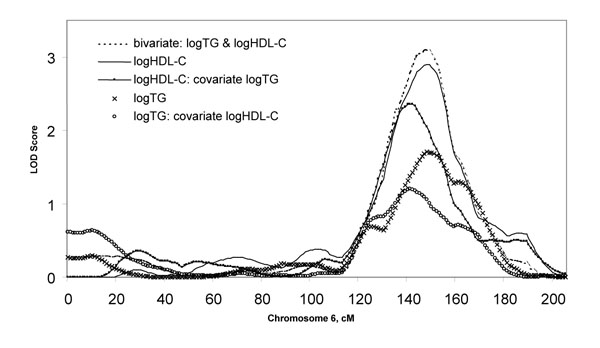
**The results of five genome scans on chromosome 6. **Multipoint LOD scores for five genome scans.

Scans 3 and 4 were of logHDL-C with and without logTG as a covariate. On scan 3, two chromosome regions were identified with maximum LOD scores equal to or greater than 1.5 (LOD = 2.6 and 2.9 on chromosome 2, at 146 cM and chromosome 6, at 149 cM, respectively). When an additional covariate, logTG, was included, the peak LOD score on chromosome 2 decreased from 2.6 to 1.7, and the peak LOD score on chromosome 6 decreased slightly from 2.9 to 2.6. This may indicate that these two regions harbor shared genes controlling the covariation for both traits, and that the shared gene on chromosome 2 may play a greater role in controlling the variation of HDL-C than that of TG, because this region was not identified by the scans of logTG. In the bivariate linkage analyses, the LOD score on the chromosome 6 region at 148 cM increased (LOD = 3.1) (Figure 1). This supports the idea that the gene in this region is involved in controlling covariation of both traits. Scan 4, the genome scan of logHDL-C with logTG as a covariate, identified one chromosomal region, chromosome 1 at 1 cM, with a maximum LOD score of 1.6. The LOD score increased compared to that of scan 3, without logTG as a covariate. This may be evidence that a putative locus specific to HDL-C may be in this region. In the bivariate linkage analyses, the LOD score in this region was decreased compared with that of scan 4. This is further evidence that the gene in this region may be related to HDL-C variation only, instead of being a shared gene.

The region on chromosome 6 with highest multipoint LOD scores in the last three genome scans is near marker D6S1009-GATA184A08. Interestingly, the same region was identified by a genome scan of TG as well as BMI, a TG and HDL-C related trait, in nondiabetic Mexican Americans [11].

## Conclusions

In conclusion, the results of our five genome scans identified chromosomal regions in which QTL may reside and that might influence one of the traits, such as HDL-C, or control the covariation of both traits, TG and HDL-C. There is strong evidence for a QTL on chromosome 6 near marker GATA184A08 appearing to influence the covariation of HDL-C and TG in the Framingham population.

## Figures and Tables

**Table 1:** The highest multipoint LOD scores equal or greater than 1.5^A^

1:logTG^B^			2: logTG^C^			3:logHDL-C^B^			4:logHDL-C^D^			5:logTG&logHDL-C^B^		
				
Chr	cM	LOD	Chr	cM	LOD	Chr	cM	LOD	Chr	cM	LOD	Chr	cM	LOD
6	148	1.7	(6	141	1.2)	(1	1	0.8)	1	1	1.6	(1	1	1.0)
7	169	1.5	(7	169	0.6)	2	146	2.6	2	141	1.7	2	146	1.7
12	3	1.5	(12	3	1.0)	6	149	2.9	6	141	2.4	6	148	3.1
20	49	2.1	(20	49	1.3)							(7	171	0.9)
												(12	3	0.7)
												(20	49	1.3)

^A^If the maximum LOD score in one scan reaches 1.5, the results of the correspondent chromosome region in another scan, with or without the second trait as a covariate (e.g., between scan 1 and scan 2 or between scan 3 and scan 4), were also reported even though the LOD scores may not reach 1.5 (in parenthesis). In the bivariate linkage analyses, in addition to those chromosome regions with LOD score 1.5 or higher, the results of other chromosome regions which have been identified in scan 1 through scan 4 are also displayed. ^B^With covariates age, sex, alcohol, and smoking. ^C^With covariates age, sex, alcohol, smoking, and logHDL-C. ^D^With covariates age, sex, alcohol, smoking, and logTG.
